# Oral Vaccination Reduces the Effects of *Lawsonia intracellularis* Challenge on the Swine Small and Large Intestine Microbiome

**DOI:** 10.3389/fvets.2021.692521

**Published:** 2021-07-16

**Authors:** Fernando L. Leite, Brittanie Winfield, Elizabeth A. Miller, Bonnie P. Weber, Timothy J. Johnson, Fred Sylvia, Erika Vasquez, Fabio Vannucci, Dana Beckler, Richard E. Isaacson

**Affiliations:** ^1^Boehringer Ingelheim Animal Health USA Inc., Duluth, GA, United States; ^2^Department of Veterinary and Biomedical Sciences, College of Veterinary Medicine, University of Minnesota, Saint Paul, MN, United States; ^3^Department of Veterinary Population Medicine, College of Veterinary Medicine, University of Minnesota, Saint Paul, MN, United States; ^4^Gut Bugs Inc., Fergus Falls, MN, United States

**Keywords:** *Lawsonia intracellularis*, oral vaccine, microbiome, swine, pathobiont

## Abstract

Porcine proliferative enteropathy remains one of the most prevalent diseases in swine herds worldwide. This disease is caused by *Lawsonia intracellularis*, an intracellular bacterial pathogen that primarily colonizes the ileum. In this study, we evaluated changes to the microbiome of the ileal mucosa, ileal digesta, cecal digesta, and feces subsequent to challenge with *L. intracellularis* and to an oral live vaccine against *L. intracellularis*. Given that gut homogenates have been used since 1931 to study this disease, we also characterized the microbial composition of a gut homogenate from swine infected with *L. intracellularis* that was used as challenge material. The *L. intracellularis* challenge led to a dysbiosis of the microbiome of both the small and large intestine marked by an increase of pathobionts including *Collinsella, Campylobacter, Chlamydia*, and *Fusobacterium*. This microbiome response could play a role in favoring *L. intracellularis* colonization and disease as well as potentially predisposing to other diseases. Vaccination altered both small and large intestine microbiome community structure and led to a significant 3.03 log_10_ reduction in the amount of *L. intracellularis* shed by the challenged pigs. Vaccination also led to a significant decrease in the abundance of *Collinsella, Fusobacterium*, and *Campylobacter* among other microbial changes compared with non-vaccinated and challenged animals. These results indicate that *L. intracellularis* infection is associated with broad changes to microbiome composition in both the large and small intestine, many of which can be mitigated by vaccination.

## Introduction

*Lawsonia intracellularis* is a Gram-negative intracellular bacterial pathogen and the causative agent of porcine proliferative enteropathy (PPE). Infection with *L. intracellularis* has been estimated to occur in more than 90% of swine farms worldwide (1, 2). *L. intracellularis* infects intestinal epithelial cells, preferentially those of the terminal ileum, and are present in cells in the crypts, causing hyperplasia leading to thickened intestinal tissue. Lesions in swine are also marked by a loss of mucin-producing goblet cells and a decrease of villus height (1, 3, 4). There are two clinical forms of PPE: porcine intestinal adenomatosis (PIA) and porcine hemorrhagic enteropathy (PHE). PHE occurs primarily in older animals, is hard to reproduce experimentally, and is associated with death and hemorrhagic diarrhea. PIA occurs in growing pigs and can lead to significant loss in production performance, diarrhea, and intestinal lesions (1, 2).

The gut microbiome is known to influence health as well as production performance of swine (5). It is also known that the composition of the microbiome varies between different portions of the intestinal tract and that the ileum is the preferential site of *L. intracellularis* infection (2, 5, 6). Research from our group has found that *L. intracellularis* can significantly alter the gut microbiome, with one of the likely consequences being the promotion of *Salmonella enterica* infection in swine (7, 8).

This study had the objective of extending the characterization of the microbiome response to *L. intracellularis* infection by evaluating microbial changes at different stages of disease, in different portions of the intestinal tract and in association with diagnostic measurements, in an attempt to better understand the underlying microbial community interactions occurring with disease. Additionally, the response to an oral live vaccine against *L. intracellularis* was investigated to determine if alterations to the microbiome of animals that were immunized were correlated with a less severe disease.

## Materials and Methods

### Animals and Experimental Design

The animal protocol used was approved by the Boehringer Ingelheim Animal Health USA Inc., Institutional Animal Care and Use Committee (protocol number 100896); and all experiments were performed in accordance with relevant guidelines and regulations. All pigs used in this study were obtained from a sow herd with no previous history of clinical signs from *L. intracellularis* infection. After being weaned, at 3 weeks of age, pigs were randomly allocated to three different treatments groups: Control (non-infected, non-vaccinated control group), Law (challenged with *L. intracellularis*), and LawVac (vaccinated and challenged with *L. intracellularis*). The LawVac group was vaccinated with the oral live vaccine (Enterisol^®^ Ileitis, Boehringer Ingelheim Animal Health USA Inc., Duluth, GA, USA) after 5 days of acclimation. To investigate the impact of the vaccine alone on the microbiome, a group of six random pigs in the Control and LawVac groups were necropsied at 21 days post-vaccination [0 days post-infection (dpi)]. At 6 weeks of age, 3 weeks post-vaccination, the Law and LawVac groups were challenged with a gut homogenate containing 4 × 10^7^ *L. intracellularis* organisms. To evaluate the microbiome response at different stages of infection, six pigs were randomly selected from each treatment group (Control, Law, and LawVac) and euthanized at 7, 21, and 28 dpi. To minimize the exposure of the Control group to *L. intracellularis*, this treatment group was housed in a separate barn from the challenged and vaccinated groups. Separation of animals within the same room has been found to be insufficient to prevent transmission of *L. intracellularis* (9). To further prevent chances of cross contamination among groups, strict biosecurity measures were followed. This included not exposing the Control group to any equipment or attire used in caring for *Lawsonia*-challenged animals. Additionally, empty pens were kept between the Law and LawVac groups to minimize the chances of contact and transmission of microorganisms between these treatment groups. All pigs were held in slatted floor pens at a density appropriate for their size. All pigs received the same feed, which was formulated by a nutritionist and did not contain antibiotics. Each treatment group comprised three pens with eight animals each, except the Law group, which had three pens with six animals each. This was because the Law treatment group was not different from the Control group at time of challenge; thus, no animals were euthanized at this time point from this treatment group. Two animals died of mulberry heart disease during the study, and samples were not collected for microbiome characterization from these animals (one pig in the Control and one pig in the LawVac treatment groups).

### Sample Collection

Since the microbiome is known to differ along the different sections of the intestinal tract of pigs (6, 10), we collected samples to represent both the large and small intestine to better characterize the intestinal microbiome. Considering that *L. intracellularis* preferentially infects epithelial cells located in the terminal ileum, we also collected mucosal scrapings from this segment as previously described, with the addition of RNAlater (Thermo Fisher Scientific, Waltham, MA, USA) (4). In addition to ileum scrapings, digesta of the terminal ileum (luminal contents) and digesta from the cecum and feces were also collected during necropsy. These samples were frozen at −80°C soon after collection. Fecal samples were also collected from all animals 5 days after allocation to treatment (−23 dpi), as well as on 0, 7, 21, and 28 dpi.

### Serology, Gross, and Microscopic Lesion Evaluation

To characterize the infection status and seroconversion of animals, blood samples were collected on all animals 5 days after allocation to treatment (−23 dpi), as well as on 0, 7, 21, and 28 dpi. Samples were tested by the immune peroxidase monolayer test at the University of Minnesota Veterinary Diagnostic Laboratory (11). The entire intestinal tract was examined for macroscopic lesions characteristic of PPE. Gross lesions were evaluated with the following rubric: 0 = no gross lesion; 1 = mild edema and hyperemia of mucosa or serosa; 2 = edema, hyperemia, and reticulated serosa and mucosa (thickening); 3 = edema, hyperemia, reticulated serosa and mucosa, and gross thickening of the mucosa; and 4 = severe thickening mucosal hemorrhaging or necrosis. A portion of the terminal ileum was collected in formalin for microscopic evaluation to determine the presence or absence of lesions. Immunohistochemistry (IHC) was used to assess the presence of *L. intracellularis*-specific antigens in the tissues as previously described (12). IHC was evaluated on a 5-point scale, as follows: 0 = absence of *Lawsonia* antigen; 1 = 0–25% of crypts with antigen; 2 = 25–50% of crypts with antigen; 3 = 50–75% of crypts with antigen; and 4 = 75–100% of crypts with antigen.

### DNA Extraction, Sequencing, and Microbiome Analysis

DNA was extracted from 250 mg of each sample using the QIAGEN DNeasy PowerSoil HTP 96 Kit (QIAGEN, Germantown, MD, USA) following the manufacturer's protocol. The resulting DNA was stored at −80°C. The V4 region of the 16S rRNA gene was amplified from DNA samples and sequenced on the Illumina, Inc. (San Diego, CA, USA) MiSeq platform (2 × 300 bp, v3 chemistry) at the University of Minnesota Genomics Center (Minneapolis, MN, USA), as previously described (13).

Raw reads were processed using the DADA2 pipeline (v2.0) (14) in R (v3.6.1) (15). Forward and reverse reads were trimmed to 200 and 160 bp, respectively, with all other parameters set to default values. Taxonomy was assigned using the SILVA small subunit rRNA sequence database (v132) (16). The resulting amplicon sequence variant (ASV) count table was filtered to remove samples with <2,500 total reads. ASVs were removed if they were classified as Archaea, Eukarya, mitochondria, chloroplasts, or unknown; had <10 reads; and/or occurred in only one sample.

For alpha diversity analyses, cecal digesta, feces, ileal mucosa, and ileal digesta samples were rarefied to 12,027, 8,156, 2,447, and 12,223 reads, respectively. ASV richness and the Shannon diversity index were calculated with the R package, phyloseq (v1.28.0) (17). To assess differences in alpha diversity between the treatment groups, non-parametric Wilcoxon rank-sum tests were performed, and *p*-values were adjusted for multiple testing using the Benjamini–Hochberg procedure.

Prior to performing beta diversity analyses and differential abundance testing, cumulative sum scaling (CSS) was used to normalize the unrarefied data using the phyloseq *phyloseq_transform_css* function. The Bray–Curtis dissimilarities and weighted UniFrac distances were then calculated using the phyloseq distance function. Differences in microbial composition between the treatment groups and dpi were visualized with principal coordinate analysis (PCoA) plots with multivariate t-distribution ellipses. Significant differences in composition between groups were assessed by permutational multivariate analysis of variance (PERMANOVA) tests with 999 permutations with the *adonis* function from the R package, vegan (v2.5.5) (18). The *betadisper* function, for PERMDISP analysis, was then used to confirm that the PERMANOVA results were not simply artifacts of heterogeneous dispersion between groups. Differential abundance testing of genus-level CSS-normalized counts between treatment groups (Law vs. Control and Law vs. LawVac) was conducted with zero-inflated Gaussian mixture models using the fitZIG function from the R package, metagenomSeq (v1.26.3) (19) with default parameters. Separate models were fit for each comparison (Law vs. Control and Law vs. LawVac) at each dpi time point (7, 21, and 28 dpi) in each intestinal segment (cecal digesta, feces, ileal mucosa, and ileal digesta). Genera occurring in only one sample were removed. Model coefficients and associated statistics, including the log2-fold changes in abundance, were viewed with the function MRcoefs. *p*-Values were adjusted for multiple comparisons using the Benjamini–Hochberg procedure. An alpha of 0.05 was used as the significance threshold in all statistical tests.

### *Lawsonia intracellularis* Inoculum Preparation, Quantification, and Metagenomic Analysis

The preparation of gut homogenate challenge material followed procedures previously described (20). Briefly, mucosal scrapings from the ileum of pigs with confirmed *L. intracellularis* infection and gross PPE lesions were homogenized and diluted in sucrose potassium glutamate diluent and Dulbecco's Modified Eagle Medium (DMEM) prior to intragastric inoculation of animals.

Quantification of *L. intracellularis* present in fecal samples, cecal digesta, and gut homogenate was performed by qPCR (21). Briefly, extraction of fecal samples was performed using the MagMAX Pathogen RNA/DNA Kit (Thermo Fisher). qPCR was performed using the Thermo Scientific VetMAX™ *L. intracellularis* PCR Kit on a Roche Diagnostics (Basel, Switzerland) 480 LightCycler per manufacturer's protocol. A standard curve was included in each run for the quantification of *L. intracellularis*. Statistical differences in fecal shedding of *L. intracellularis* were assessed by Wilcoxon's rank-sum test.

With the objective of gaining a broad understanding of the bacterial and viral taxa of the gut homogenate inoculum material, shotgun metagenomic analysis was performed. Metagenomic characterization of the gut homogenate challenge material was performed at Newport Laboratories (Worthington, MN, USA). DNA and RNA extraction was performed using MagMax Core Nucleic Acid Purification Kit (Thermo Fisher). cDNA was synthesized from RNA with the Maxima H Minus Double-Stranded cDNA Synthesis Kit (Thermo Fisher). Library preparation was performed with the Nextera XT kit (Illumina), and sequencing was performed on MiSeq sequencer with 2 × 300 bp V3 chemistry (Illumina). Sequence read files were uploaded on to Illumina BaseSpace for analysis. Scaffolds were created using Illumina BaseSpace application SPAdes Genome Assembler. Metagenomic analysis was performed using Illumina BaseSpace application Kraken (v1.0.0) (22). The generated summary report was filtered for virus and bacterial species detected.

## Results

### Composition of Gut Homogenate Challenge Material

Shotgun metagenomic analysis of the gut homogenate challenge material revealed the presence of 66 different bacterial species among 164,123 sequencing reads. The most abundant was *L. intracellularis*, which represented approximately 0.40% of total reads and 34.9% of bacterial reads (Supplementary Table 1). Other bacteria present included *Campylobacter fetus, Campylobacter jejuni, Campylobacter concisus, Chlamydia trachomatis, Bacteroides fragilis*, and *Fusobacterium nucleatum*. At a much lower abundance, sequences matching eight different viruses were also detected in the gut homogenate and included porcine type C oncovirus (Supplementary Table 2).

### Measures of *Lawsonia intracellularis* Infection and Shedding Over Time

**Figure 1:**
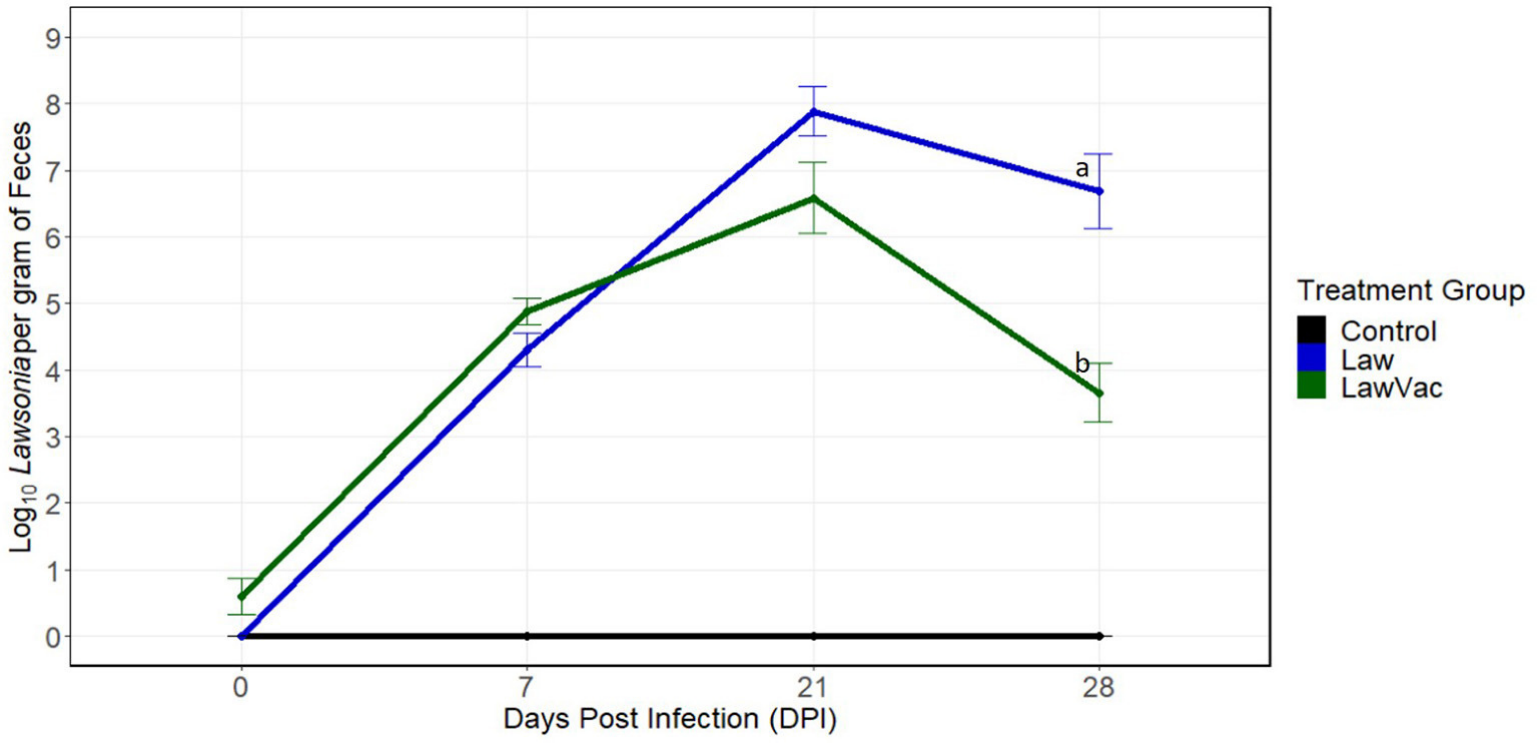
Quantification of *Lawsonia intracellularis* shedding in fecal samples over time, measured by qPCR. Mean and standard error of the mean at 0 days post-infection (*n* = 18 pigs per treatment group), 7 days post-infection (*n* = 18 pigs per treatment group), 21 days post-infection (*n* = 12 pigs per treatment group), and at 28 days post-infection (*n* = 6 pigs per treatment group). Different letters indicate statistical significance [Wilcoxon rank-sum test (*p* < 0.05)].

To better understand how the composition of the gut microbiome changed during infection with *L. intracellularis*, sequential necropsies were performed at 0, 7, 21, and 28 days post-challenge. First, shedding of *L. intracellularis* was measured at the various time points. Fecal shedding was evaluated at 0 and 7 dpi among 18 pigs per group and at 21 and 28 dpi among 12 and six pigs per group, respectively. Shedding of *L. intracellularis* was not detected in the feces of pigs in the Control group and the group challenged with *L. intracellularis* at 0 dpi. In the challenged (“Law”) group, shedding increased from 4.3 log_10_ organisms per gram at 7 dpi to 7.87 log_10_ organisms per gram at 21 dpi and then decreased to 6.69 log_10_ organisms per gram at 28 dpi (Figure 1). Pigs in the vaccinated and challenged (“LawVac”) group had on average 0.60 log_10_ *L. intracellularis*/gram in feces at 0 dpi. Since the pigs at 0 dpi had not been challenged yet, detectable *L. intracellularis* likely was from the live oral vaccine strain. At 21 dpi, the LawVac group shed on average 6.59 log_10_ organisms per gram and at 28 dpi, 3.66 log_10_, the latter representing a 3.03 log_10_ reduction in the shedding of *L. intracellularis* (*p* = 0.008) as compared with non-vaccinated Law pigs (Figure 1). Both Law and LawVac groups had positive IHC staining of *L. intracellularis* at 7, 21, and 28 dpi. The LawVac group did not have positive IHC staining at 0 dpi and had less severe microscopic lesions at 28 dpi. At this time point, the LawVac group had a median lesion score of 0.5 with three of six (50%) pigs with positive IHC staining, while the Law group had a median lesion score of 2 with five of six pigs (83%) with positive IHC staining. While both Law and LawVac groups had macroscopic lesions at 21 dpi, only the Law group had macroscopic lesions characteristic of *L. intracellularis* infection at 28 dpi with two of six (33%) pigs having a lesion score of 2 and 4, respectively. No macroscopic lesions were found in the LawVac group at this time point. No macroscopic lesions were found at 0 or 7 dpi. At 3 weeks post-vaccination, only one of the 24 (4.2%) LawVac animals had serum antibodies against *L. intracellularis*. This proportion increased to 23.5% (4/17), 90.9% (10/11), and 83.3% (5/6) at 7, 21, and 28 dpi, respectively. All Law animals were seronegative at 0 dpi, and seroconversion increased to 33.3% (6/18), 63.6% (7/11), and 100% (6/6) of pigs with serum antibodies against *L. intracellularis* at 7, 21, and 28 dpi, respectively. Control animals remained negative for all diagnostic measures of *L. intracellularis* infection throughout the trial.

### *Lawsonia intracellularis* Infection Alters Microbial Community Structure of the Small and Large Intestine

**Figure 2:**
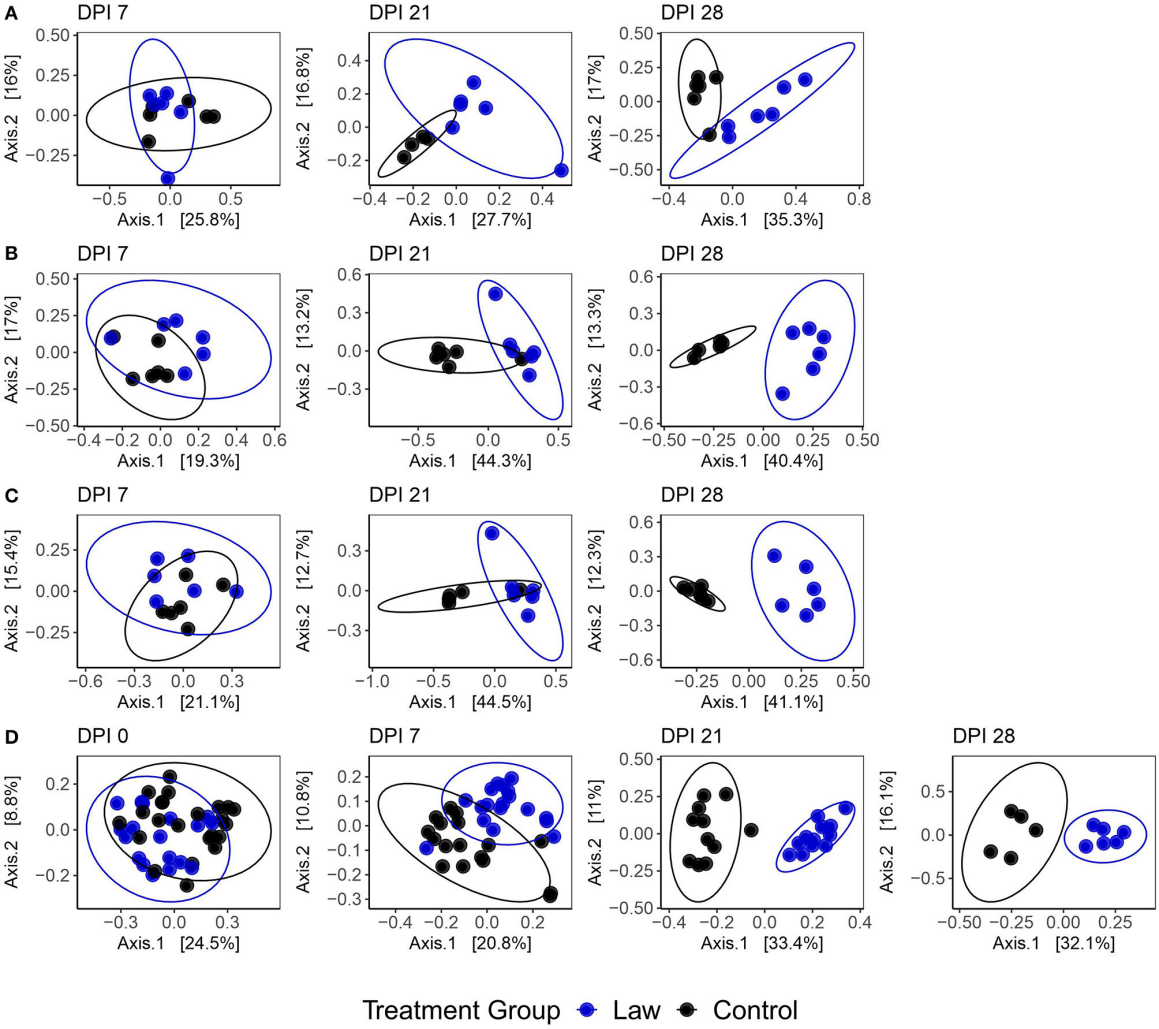
Principal coordinate analysis plots using the Bray–Curtis dissimilarity of samples from **(A)** ileal mucosa; **(B)** ileal digesta; **(C)** cecal digesta; and **(D)** feces among Law and Control groups at different days post-infection (dpi). Ellipses represent the 95% confidence level of the multivariate Student's *t*-distribution.

Sequencing of the 16S rRNA gene for microbiome analysis generated mean reads per sample of 23,599 ± 5,059, 21,645 ± 4,831, 16,639 ± 6,742, and 21,938 ± 3,748 for the cecal digesta, feces, ileal mucosa, and ileal digesta, respectively. To determine if changes in microbiome community composition were altered by infection with *L. intracellularis*, a beta diversity analysis was performed among the challenged and non-challenged Law and Control groups utilizing the Bray–Curtis dissimilarity index. A clear effect of challenge on community structure was observed. Figure 2 shows a clear segregation between Law and Control treatments that becomes more evident as infection progresses. At the site of *L. intracellularis* infection, the terminal ileal mucosa, no significant differences were observed at 7 dpi (PERMANOVA: *p* = 0.43, *R*^2^ = 0.082; Supplementary Table 3), while a clear change in community structure was observed at 21 dpi (*p* = 0.0010, *R*^2^ = 0.21) and 28 dpi (*p* = 0.0070, *R*^2^ = 0.28; Figure 2A). Among ileal digesta, a significant difference was observed at 7 dpi (*p* = 0.046, *R*^2^ = 0.13), but similar to ileal mucosa, differences also increased as infection progressed at 21 dpi (*p* = 0.011, *R*^2^ = 0.31) and 28 dpi (*p* = 0.0040, *R*^2^ = 0.380; Figure 2B). Among fecal samples (Figure 2D), while at 0 dpi, a significant difference among groups was detected (*p* = 0.0010, *R*^2^ = 0.078); this difference was not present at −23 dpi (*p* = 0.36, *R*^2^ = 0.025). Treatment group explained a drastically increased proportion of microbial community variation as infection progressed, with PERMANOVA *R*^2^-values of 0.095, 0.31, and 0.30 at 7, 21, and 28 dpi, respectively (all *p* ≤ 0.005). Finally, a similar trend to other communities was observed in the cecal digesta, with no significant differences observed at 7 dpi (*p* = 0.13, *R*^2^ = 0.12) and an increase in the distinction between treatment group microbial communities as infection progressed at 21 dpi (*p* = 0.0050, *R*^2^ = 0.31) and 28 dpi (*p* = 0.0040, *R*^2^ = 0.41; Figure 2C). All tests for homogeneity of variance between sample groups were non-significant, providing support for true differences in composition between dpi. Similar results were also observed when analyses were conducted using weighted UniFrac distance instead of Bray–Curtis dissimilarity (Supplementary Figure 1 and Supplementary Table 3). Together, these findings indicate broad changes to community composition due to *L. intracellularis* challenge, which progress during infection in both the small and large intestine.

Changes to alpha diversity were not as evident as those found in beta diversity. Among fecal samples, Shannon's diversity index was significantly higher in the Law group compared with the Control group at 0 and 7 dpi (Supplementary Figure 2A). For the number of ASVs, the Law group was higher than the Control group at −23 dpi, but this difference was not subsequently observed at 0 dpi through 28 dpi (Supplementary Figure 2B). No significant differences in the Shannon diversity index or number of ASVs were found in ileal mucosa, ileal digesta, and cecal digesta between the Law and Control groups (data not shown).

### Differential Abundance in *Lawsonia intracellularis* Infected Pigs in Different Intestinal Segments

**Figure 3:**
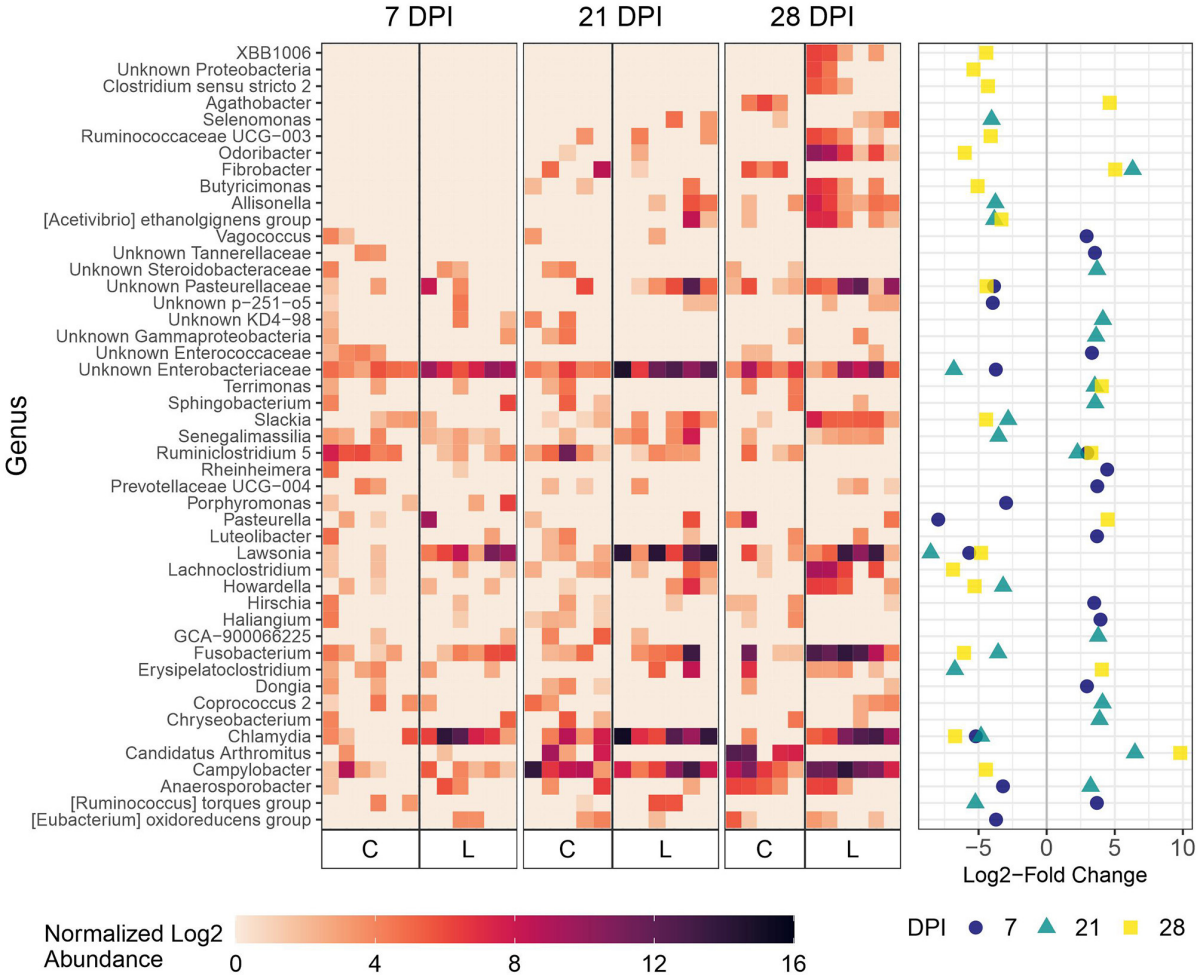
Differential abundance of microbial genera in ileal mucosa between the Law (L) and Control (C) groups at 7, 21, and 28 dpi. The 20 genera with the largest significant log2-fold change at each dpi are included. The heatmap shows the cumulative sum scaling (CSS)-normalized log2 abundance of each genera per sample, with the significant log2-fold changes by dpi plotted on the right panel. Note that a genus only has to be one of the top 20 significantly differentially abundant at 1 dpi for it to be included in the figure. A positive log2-fold change indicates greater abundance in the Control group, and a negative log2-fold change indicates greater abundance in the Law group.

**Figure 4:**
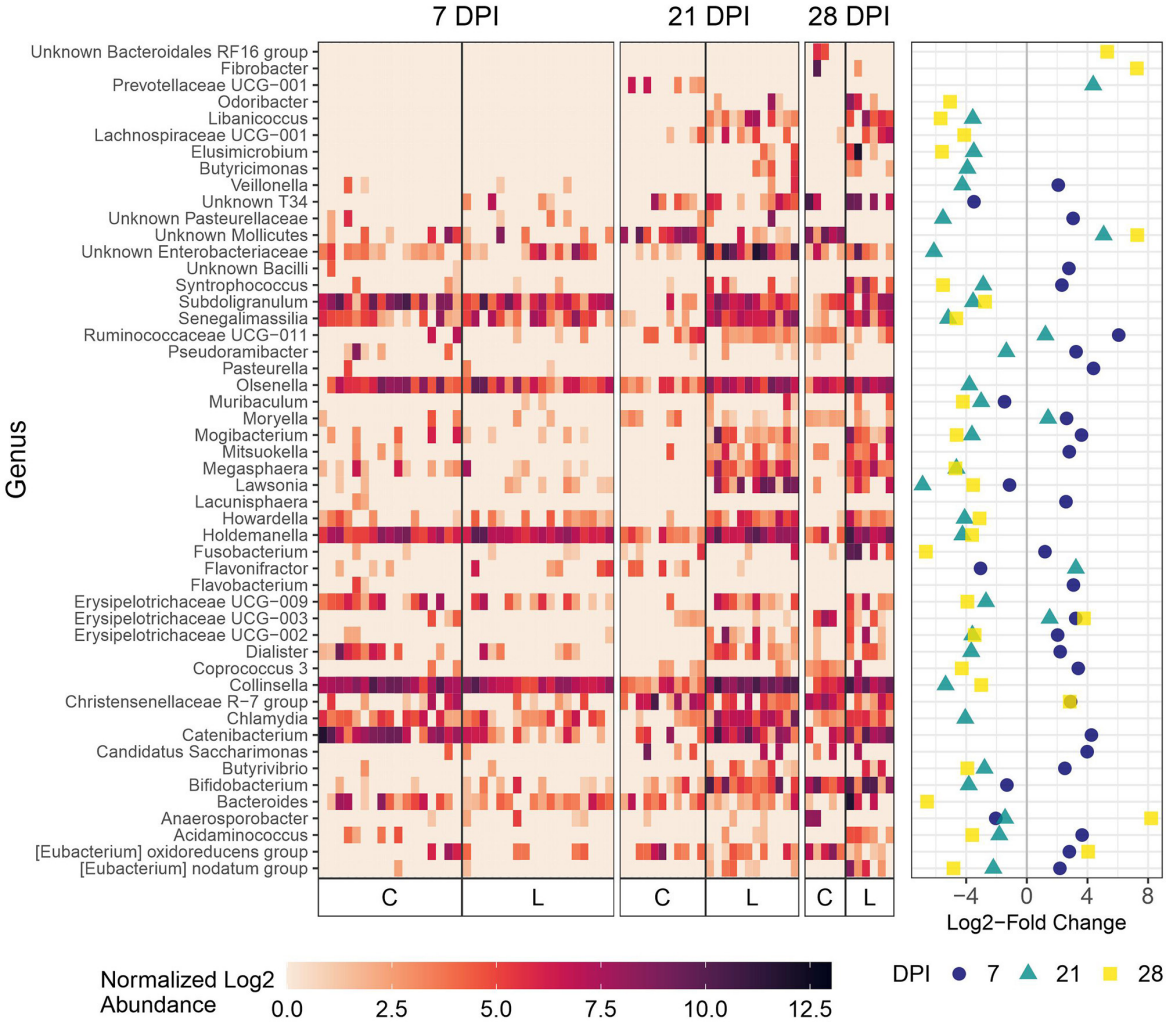
Differential abundance of microbial genera in feces between the Law (L) and Control (C) groups at 7, 21, and 28 dpi. The 20 genera with the largest significant log2-fold change at each dpi are included. The heatmap shows the cumulative sum scaling (CSS)-normalized log2 abundance of each genera per sample, with the significant log2-fold changes by dpi plotted on the right panel. Note that a genus only has to be one of the top 20 significantly differentially abundant at 1 dpi for it to be included in the figure. A positive log2-fold change indicates greater abundance in the Control group, and a negative log2-fold change indicates greater abundance in the Law group.

As expected, microbial composition differed between the intestinal segments sampled (Supplementary Figure 3). The *Lawsonia* genus was detected by 16S rRNA gene analysis and found to be significantly differentially abundant between the Control and Law groups. Following a similar trend to that observed by qPCR quantification, the greatest differential abundance of *Lawsonia* was found at 21 dpi, at which point *Lawsonia* was the most differentially abundant genus among the Control and Law groups in both feces and ileal mucosa samples (Figures 3, 4).

At 21 dpi, *Lawsonia* was significantly more abundant in the ileal mucosa of pigs in the Law group compared with Control (Figure 3). *Chlamydia, Fusobacterium*, and unknown members of the family Enterobacteriaceae were also significantly more abundant (Figure 3). Several genera were more abundant in Control animals at this time point compared with Law pigs, including *Candidatus* Arthromitus, *Fibrobacter*, and *Ruminiclostridium 5* (Figure 3). At 7 dpi, *Lawsonia, Chlamydia*, and unknown Enterobacteriaceae were also more abundant in the Law group (Figure 3). At 28 dpi, along with *Lawsonia* and *Chlamydia, Campylobacter* along with more genera were more abundant in the Law group (Figure 3).

In the ileal digesta, only two genera were differentially abundant between Law and Control pigs at 7 dpi: *Slackia* and *Catenibacterium* both being more abundant in Control animals (*p* < 0.01). Interestingly, at both 21 and 28 dpi, *Slackia* was more abundant in Law than Control pigs (*p* < 0.01; Supplementary Figure 4). At 21 dpi, *Campylobacter* was more abundant in the Law than Control pigs, along with *Lawsonia* and *Collinsella*, among others. At 28 dpi, again *Collinsella* was among the most differentially abundant genus in Law pigs compared with Control pigs along with *Lawsonia* (Supplementary Figure 4).

Similar to ileal digesta, in cecal digesta, only few genera were differentially abundant between the Law and Control groups at the initial stage of infection at 7 dpi. Again, *Slackia* along with *Catenibacterium* was differentially abundant and more abundant in Control than Law pigs (*p* < 0.05) at this time point. At 21 dpi, many more differentially abundant bacteria were found; among those most abundant in Law pigs were *Lawsonia, Chlamydia, Collinsella*, and *Fusobacterium* (Supplementary Figure 5). At 28 dpi, even more bacteria were differentially abundant; in addition to those seen at 21 dpi, *Bacteroides* among others were more abundant in the Law group than the Control group (Supplementary Figure 5).

At 21 days dpi when *L. intracellularis* was at the highest levels in feces as measured by qPCR, differential abundance was detected for *Lawsonia* and several other bacteria including *Chlamydia, Collinsella*, and unknown Enterobacteriaceae (Figure 4) in challenged animals compared with non-challenged Control animals. At 28 dpi, the two most differentially abundant genera with the greatest abundance in the Law group compared with the Control group were *Fusobacterium* and *Bacteroides*, while some of the same bacteria differentially abundant at 21 dpi were found at 28 dpi including *Collinsella*. At the initial stage of infection (7 dpi), *Lawsonia* was already significantly differentially abundant among groups in feces (Figure 4). Similar to ileal and cecal digesta, *Catenibacterium* was more abundant in Control animals at this time point (Figure 4).

**Figure 5:**
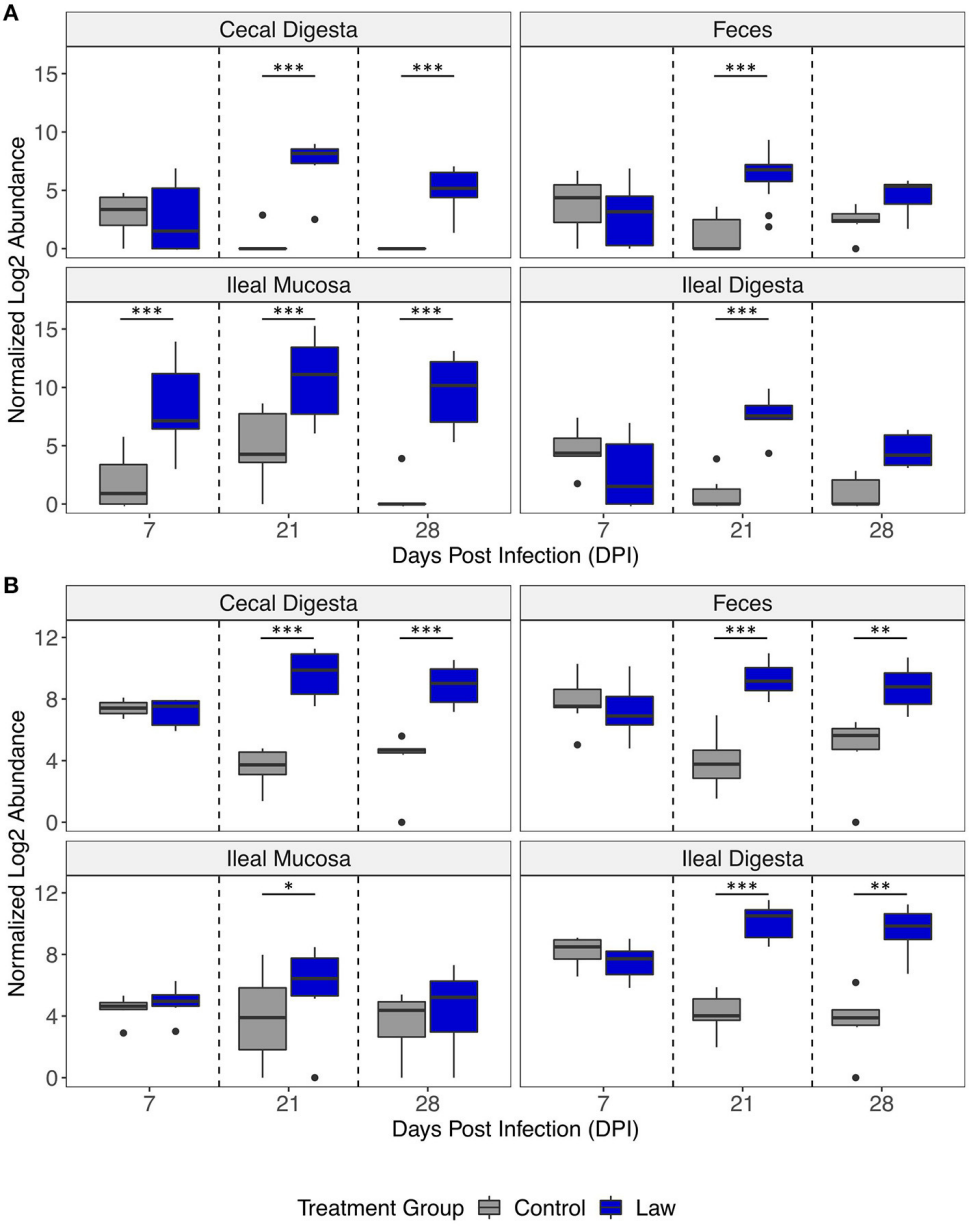
Differential abundances of the genera **(A)** *Chlamydia* and **(B)** *Collinsella* between Law and Control treatment groups in different intestinal segments over days post-infection. Asterisks indicate significant differences between treatment groups. *p < 0.05; **p < 0.01; and ***p < 0.001.

The differential abundances of *Chlamydia* and *Collinsella* between treatment groups are shown in Figure 5. In all time points post-infection, *Chlamydia* was significantly more abundant in the ileal mucosa of the Law group, while only at 21 dpi was it significantly greater in abundance in ileal digesta and feces (Figure 5A). For *Collinsella*, only at 21 dpi it was found to be significantly more abundant in ileal mucosa, while at both 21 and 28 dpi, it was significantly more abundant in feces and ileal digesta of Law pigs (Figure 5B). This further highlights the differential abundance observed between Law and Control animals in the different intestinal segments and time points of infection.

### Microbiome Changes Induced by Vaccination

An oral live vaccine has been used to control *L. intracellularis* infection since 2001, and while its effect on the microbiome has been previously described (8), we wanted to extend these findings to investigate different intestinal segments and time points as well as focus on its impact to *L. intracellularis* challenge. At 3 weeks post-vaccination (0 dpi), a significant difference in community structure was found between LawVac and Law pigs in feces (Bray–Curtis dissimilarity PERMANOVA: *p* = 0.042, *R*^2^ = 0.041; Supplementary Table 4), while no differences in community structure were found between groups prior to vaccination at −23 dpi (*p* = 0.41, *R*^2^ = 0.025). Vaccination had a significant effect (*p* < 0.05) on the community structure of ileal mucosa, ileal digesta, and cecal digesta relative to non-vaccinated Control pigs at 0 dpi (data not shown). Investigating composition further, at 0 dpi, there were 73 significantly differentially abundant genera between LawVac and Law pigs in feces (Supplementary Table 5).

During infection, at 7, 21, and 28 dpi, fecal community structure was also significantly different between the LawVac and Law groups. An increase in composition dissimilarity between groups was observed with the progression of infection from 7 dpi (*p* = 0.023, *R*^2^ = 0.054) to 28 dpi (*p* = 0.061, *R*^2^ = 0.14; Supplementary Figure 6D). Among cecal digesta, a significant difference in community structure was only observed at 28 dpi (*p* = 0.024, *R*^2^ = 0.16; Supplementary Figure 6C). While no significant differences in community structure were noted in ileal digesta between the Law and LawVac groups, they did appear as distinct groups at 28 dpi (*p* = 0.067, *R*^2^ = 0.14; Supplementary Figure 6B). Among ileal mucosa, a significant difference in community structure was observed at both 7 dpi (*p* = 0.024, *R*^2^ = 0.17) and 28 dpi (*p* = 0.0070, *R*^2^ = 0.20) time points between the LawVac and Law groups (Supplementary Figure 6A). Again, there was no significant heterogeneous dispersion between sample groups and similar ordination plots; and PERMANOVAs were observed when analyses were conducted using weighted UniFrac distance instead of Bray–Curtis dissimilarity (Supplementary Figure 7 and Supplementary Table 4).

**Figure 6:**
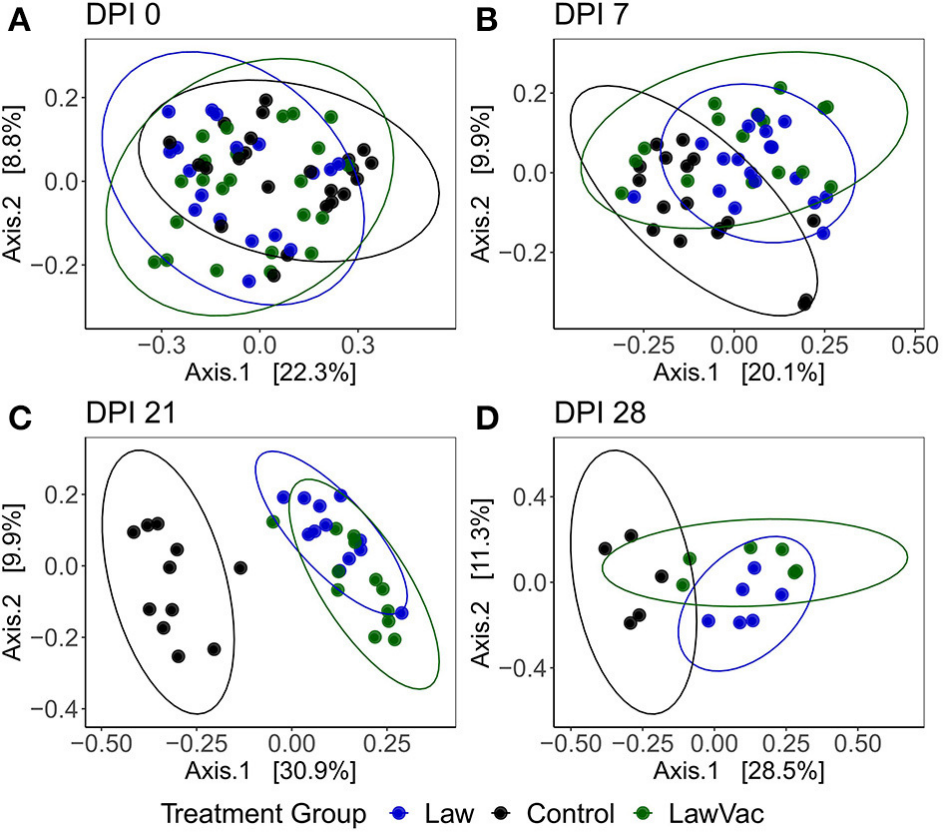
Principal coordinate analysis plots using the Bray–Curtis dissimilarity of fecal microbiota among Control, Law, and LawVac groups at **(A)** 0 days post-infection; **(B)** 7 days post-challenge; **(C)** 21 days post-infection; and **(D)** 28 days post-infection. Ellipses represent the 95% confidence level of the multivariate Student's *t*-distribution.

When comparing all treatment groups together, it was also observed that as infection progressed, so did the fecal microbial community differences among groups. While at 0 and 7 dpi, there was no clear visual distinction between treatments, at 21 dpi, both groups challenged with *L. intracellularis* were noticeably different from Control pigs (*p* = 0.0010, *R*^2^ = 0.32); and at 28 dpi, each group had a distinct community structure (*p* = 0.001, *R*^2^ = 0.30; Figure 6).

**Figure 7:**
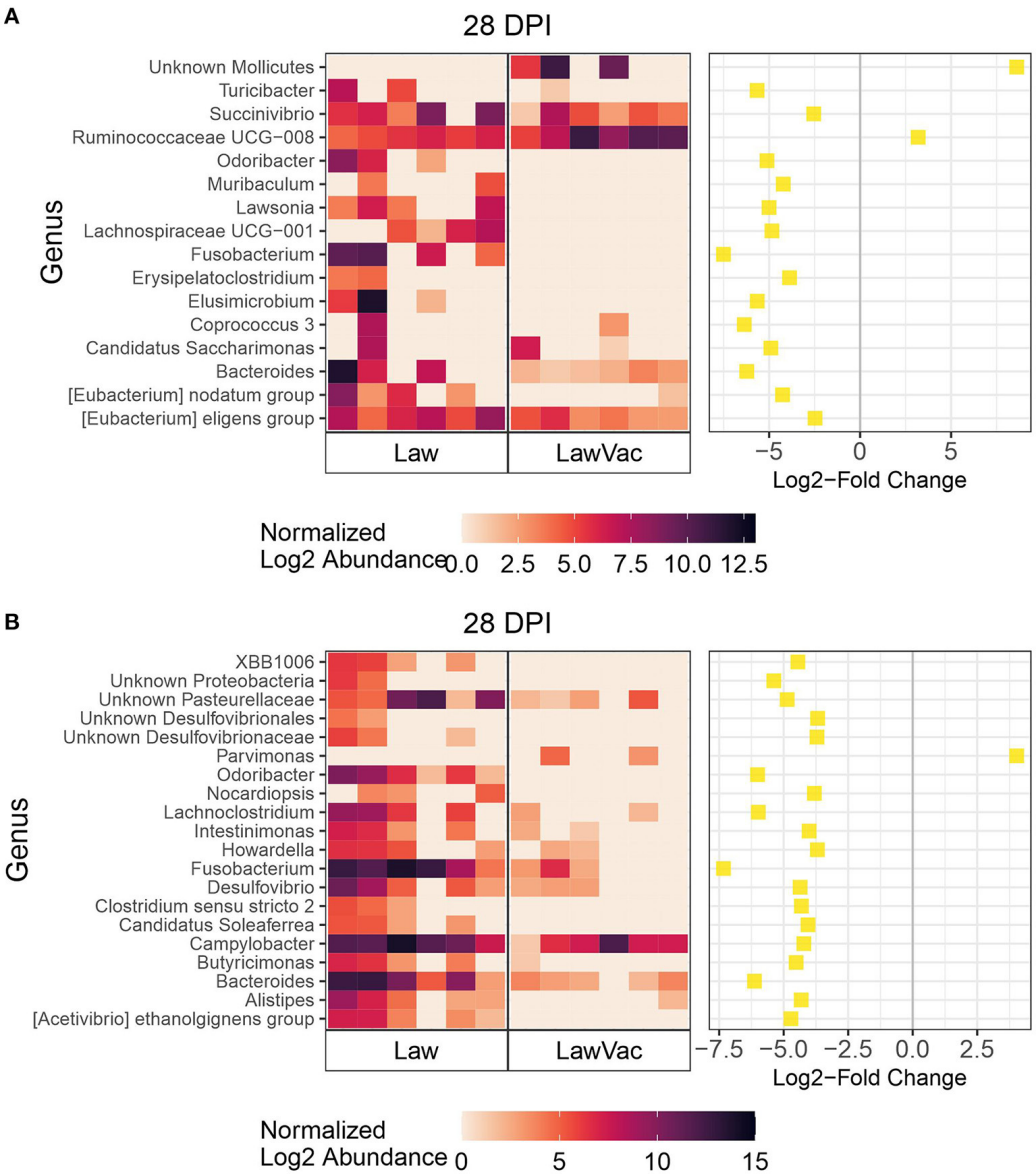
Differential abundances of microbial genera between the Law and LawVac groups at 28 dpi in **(A)** fecal samples and **(B)** ileal mucosa samples. The 20 genera with the largest significant log2-fold change are included for ileal mucosa samples. The heatmap shows the cumulative sum scaling (CSS)-normalized log2 abundance of each genera per sample, with the significant log2-fold changes plotted on the right panel. A positive log2-fold change indicates greater abundance in the LawVac group, and a negative log2-fold change indicates greater abundance in the Law group.

We then focused on the 28 dpi time point since this was the time point when vaccinated animals exhibited a significant 3.03 log_10_ reduction in the shedding of *L. intracellularis* (*p* = 0.008; Figure 1). Among fecal microbiota, only two genera were more abundant in the LawVac group in comparison with the Law group: Ruminococcaceae UCG-008 and Unknown Mollicutes (Figure 7A). *Lawsonia, Fusobacterium*, and *Bacteroides* among other genera were significantly more abundant in the Law group compared with the LawVac group at this time point (Figure 7A). In ileal mucosa samples, *Fusobacterium* and *Bacteroides* were the two most significantly abundant genera in the ileal mucosa of non-vaccinated pigs along with *Campylobacter* (Figure 7B). Only eight genera were differentially abundant in ileal digesta. While unknown *Mollicutes* were significantly more abundant in the vaccinated group, *Bacteroides* and *Fusobacterium* were significantly more abundant in the Law group (Supplementary Figure 8). Among cecal digesta, *Lawsonia* along with *Fusobacterium, Campylobacter*, and *Bacteroides* among others were significantly more abundant in non-vaccinated challenged pigs (Supplementary Figure 9).

## Discussion

This study characterized the porcine intestinal microbiome response of pigs to a *L. intracellularis* gut homogenate challenge and vaccination with an oral live vaccine. In the first description of PPE by Biester and Schwarte (23), it was noted that disease could be reproduced by feeding healthy swine intestinal contents and scrapings from affected pigs. Since then, gut homogenates, which comprise homogenate intestinal material from pigs with gross PPE lesions, have been described and used extensively to study *L. intracellularis* infection (4, 20, 24, 25). In this study, we used shotgun metagenomic sequencing to investigate the bacterial and viral composition of *L. intracellularis* gut homogenate challenge material. While *L. intracellularis* was the predominant bacterium found in the gut homogenate, other bacteria were detected along with some viruses. The relative abundance of *L. intracellularis* among other bacteria found by shotgun sequencing was similar to that found in a previous report by 16S rRNA gene sequencing, with relative abundances of 34.9% and 37.5%, respectively (26).

When analyzing the challenge dose of *L. intracellularis* provided to pigs (7.60 log_10_ *L. intracellularis* organisms), it was clear that infection and proliferation of *L. intracellularis* occurred, as *Lawsonia*-infected animals shed on average 7.87 log_10_ organisms/gram at 21 dpi. Along with detection by quantitative real-time PCR, 16S rRNA gene analysis also confirmed the presence of *Lawsonia*, which was significantly differentially abundant between Control non-infected and Law infected groups in feces, ileal mucosa, ileal digesta, and cecal digesta. Typical macroscopic lesions and positive IHC staining for the presence of *L. intracellularis* in terminal ileum tissue were also observed in these animals.

Past studies have found an intriguing relationship between *L. intracellularis* infection and gut microbiome composition. This study is not the first description of bacteria other than *Lawsonia* being enriched in the intestine of pigs challenged with a gut homogenate. McOrist et al. (24) studied gnotobiotic pigs challenged with a gut homogenate, and similar to the present study, they also found that species of *Campylobacter* and *Bacteroides* were present in the homogenate challenge material and enriched in the challenged pigs that developed PPE lesions. While we identified a low number of viruses in the gut homogenate material, McOrist et al. (24) did note that gut homogenate material was not fully infective after being more extensively filtered, suggesting that bacteria were the primary cause of disease. This indicates that underlying microbial interactions occur during infection and could contribute to development of lesions and disease.

Previous work from our research group has found that *L. intracellularis* challenge using a pure culture in conventionally raised specific pathogen free pigs does lead to a significant change in the microbial community structure sampled in cecal mucosa, colon mucosa, and feces of pigs (7, 8). In the present study, we also observed that *L. intracellularis* challenge with the gut homogenate led to a very clear and dramatic change in microbial community structure not only of the cecum and feces but also of the ileal mucosa and digesta. The fact that the differences in community structure found in Law challenged pigs increased as the infection progressed is further evidence of the interaction of *L. intracellularis* with the microbiome. While the evidence supports the hypothesis that *L. intracellularis* is the cause of disease, it is not possible to rule out the interaction of one or more additional bacteria required for disease.

When evaluating the differential abundance of various microbes due to *L. intracellularis* challenge, 16S rRNA gene analysis was used to confirm that *Lawsonia* was present in the terminal ileal mucosa, the site known to be the preferential niche of infection (1, 2). At all time points post infection, in addition to *Lawsonia, Chlamydia* and unknown members of the family Enterobacteriaceae also were more abundant in the ileal mucosa of pigs in the Law group. *Chlamydia* is a Gram-negative intracellular bacteria that can replicate in mucosal epithelial cells, including those of the gastrointestinal tract, and can cause enteritis (27). *Chlamydia* was also detected in the gut homogenate challenge material, indicating it was present in the pigs with PPE lesions used to make the challenge material and might have been the source of *Chlamydia* in the pigs in the Law group.

Among the bacteria significantly less abundant in the ileal mucosa of challenged animals were *Ruminiclostridium* 5, *Fibrobacter*, and *Candidatus* Arthromitus. The latter is a candidate genus of segmented filamentous bacteria, which are known to bind to the surface of the ileal epithelium and have the capacity of modulating immune response (28). In ileal digesta and cecal digesta, bacteria of the *Ruminiclostridium* genus were also less abundant in the challenged pigs. Interestingly, *Ruminiclostridium* abundance in fecal samples has recently been shown to be significantly and inversely correlated to *Mycoplasma hyopneumoniae* abundance in infected swine and are known to produce butyrate (29). Both *L. intracellularis* and *M. hyopneumoniae* are prevalent bacterial pathogens known to cause significant production losses, including during co-infection even when pigs are not exhibiting clinical signs (30).

At 28 dpi when Law pigs still had macroscopic lesions, *Fusobacterium* and *Bacteroides* among other bacteria were more abundant in the ileal mucosa of pigs in the Law group. *Fusobacterium* and *Bacteroides*, including *F. nucleatum* and *B. fragilis*, were also found in the gut homogenate material. *Fusobacterium* and *F. nucleatum* abundance in colonic mucosa have been found to be significantly more abundant in patients with adenoma and colorectal cancer, and these bacteria have the capacity to induce severe TNF-mediated inflammation (31, 32). Interestingly, gene expression pathways observed in intestinal tissue of pigs with PPE lesions have been found to be associated with a cellular proliferation and inflammatory signature resembling those of cancer and other inflammatory diseases of the intestinal tract (4, 33). PPE of course is not a cancer, as lesions do resolve. Similar to *F. nucleatum, Bacteroides*, and *B. fragilis* also have been implicated in colorectal cancer, in which certain strains have been found to be capable of promoting cellular proliferation by stimulation of Wnt signaling. The latter is a signaling pathway that has also been described to be altered in pigs with PPE (4, 34–36). Increased abundance of *Bacteroides* has also been found in the fecal microbiome of pigs challenged with a pure culture of *L. intracellularis* and to be negatively associated with weight gain in swine (8, 10). Considering one of the hallmarks of PPE lesions is the loss of goblet cells and the protective intestinal mucin layer (1, 37), it is likely that bacteria other than *L. intracellularis* could contribute to the development of lesions as they would have increased access to the epithelium. Additionally, mucin is known to harbor antimicrobial peptides, which, if altered during infection, could also contribute to dysbiosis (38, 39).

In this study, we also evaluated the response of pigs to an oral live vaccine against *L. intracellularis*. We observed that this vaccine led to a 3.03 log_10_ reduction in the shedding of *L. intracellularis* at 28 dpi, when LawVac pigs shed on average 3.66 log_10_ organisms per gram. *L. intracellularis* shedding level post-challenge, at levels above 6 log_10_ in particular, have a significant positive correlation to intestinal lesion severity and a significant negative correlation to average daily weight gain (25). When evaluating differential abundance at the time point when vaccinated animals shed significantly less *L. intracellularis*, several of the bacteria that were found to be more abundant in infected animals were decreased in the vaccinated group. These included *Fusobacterium, Campylobacter*, and *Bacteroides*. We have previously found that vaccination against *L. intracellularis* led to a decrease in the abundance of *Collinsella* and *Prevotella* in pigs co-infected with *L. intracellularis* and *S. enterica* serovar Typhimurium. Although not among the genera with the greatest differential abundant fold change, vaccinated pigs did have a significant (*p* < 0.05) decrease in abundance of *Collinsella* in cecal digesta and of *Prevotella* 2 in ileal mucosa (data not shown). Again, similar to previous findings, several of the sequences of *Collinsella* were closely related those of *Collinsella aerofaciens* and sequences of *Prevotella* to *Prevotella copri*. These have been considered pathobiont bacteria, as they have the capacity to induce inflammatory responses, which could favor *Salmonella* infection (40, 41). The decrease in abundance of *Collinsella* and *Prevotella* by vaccination was also previously associated with a significant 2.12 log_10_ reduction in the shedding of *S*. Typhimurium (8). This suggests that one of the consequences of *L. intracellularis* infection on microbiome composition could be increased susceptibility to other pathogens, an effect that can be minimized by vaccination. These observations suggest that oral vaccination not only can mitigate traditional measures of disease, such as reduction of lesions and shedding, but also can prevent some of the negative effects of pathogen challenge over microbiome composition, being a tool to protect this important community. The resulting microbiome could also be involved in reducing susceptibility to other pathogens, decreasing the shedding of *L. intracellularis*, decreasing PPE lesions, and improving production performance, which have been shown in studies evaluating this vaccine (8, 42–45).

It is remarkable that a vaccine that only contains antigens to one bacterium (*L. intracellularis*) also altered the abundance of other bacterial members of the gut microbiome. Perhaps this indicates that *L. intracellularis* is the primary causative agent of PPE and driver of dysbiosis, which when controlled with an effective mucosal immune response allows for minimizing its effects on microbial community composition. It is also possible but unclear if the changes observed in the gut microbiome by the oral vaccine alone or prior to challenge had any effect in the protection conferred to *L. intracellularis*.

Dysbiosis has been broadly defined as any change to the composition of resident commensal communities relative to the community found in healthy individuals. Dysbiosis can be further categorized into to three types: (i) loss of beneficial microbial organisms, (ii) expansion of pathobionts, and (iii) loss of overall microbial diversity (46). Pathobionts are bacteria that reside in the host and, although not considered primary pathogens, have the capacity to induce and contribute to disease (40, 46). This study suggests that *L. intracellularis* challenge induces dysbiosis not only due to changes to the composition of community commensal bacteria of the small and large intestine but also by an increase of pathobionts such as *Campylobacter, Chlamydia, Fusobacterium, Bacteroides*, and *Collinsella*. These effects may be mitigated by vaccination, which in this study not only significantly decreased the abundance of *L. intracellularis* but also led to a different community structure and a decreased abundance of several pathobionts induced by challenge.

Our findings support the strong association of *L. intracellularis* with other bacteria present in the swine gut that could contribute to the development and severity of disease, many of which were found to be altered by vaccination in this study. Future studies should focus on investigating microbiome changes induced by *L. intracellularis* in natural field conditions as well as utilizing gnotobiotic models with different defined microbial communities to allow for more conclusions on the exact contribution of organisms other than *L. intracellularis* in the establishment and severity of PPE.

## Data Availability Statement

The sequencing datasets generated for this study can be found in the NCBI BioProject repository under ID PRJNA728719, https://www.ncbi.nlm.nih.gov/bioproject/728719.

## Ethics Statement

The animal study was reviewed and approved by the Boehringer Ingelheim Animal Health USA Inc., Institutional Animal Care and Use Committee and all experiments were performed in accordance with relevant guidelines and regulations.

## Author Contributions

FL and RI conceived the study. BW, EM, BPW, and TJ conducted microbiome analysis. FS conducted metagenomic analysis of gut homogenate. EV, DB, and FV conducted pathological measurements and collected samples. FL, RI, and TJ wrote the manuscript. All authors have read and critically reviewed the manuscript.

## Conflict of Interest

This study was partially funded by Boehringer Ingelheim Animal Health USA Inc., Duluth, GA. FL and FS are employees of Boehringer Ingelheim Animal Health USA Inc. DB is employed by Gut Bugs Inc. The remaining authors declare that the research was conducted in the absence of any commercial or financial relationships that could be construed as a potential conflict of interest.
