# Supplementary material for: Oral Vaccination Reduces the Effects of Lawsonia intracellularis Challenge on the Swine Small and Large Intestine Microbiome
Source: Front Vet Sci. 2021 Jul 16;8:692521. doi: 10.3389/fvets.2021.692521 (PMC8322526; doi:10.3389/fvets.2021.692521)
Supplement: Supplementary file 1 [file Data_Sheet_1.pdf]

Supplementary Material

Supplementary Table 1. Bacterial species found in metagenomic characterization of *Lawsonia intracellularis* gut homogenate challenge material.

% of Total Reads	% of Bacterial Reads	Species
0.399%	34.89%	*Lawsonia intracellularis*
0.214%	18.75%	*Streptococcus suis*
0.184%	16.07%	*Alteromonas macleodii*
0.073%	6.35%	*Bacteroides fragilis*
0.038%	3.29%	*Campylobacter fetus*
0.031%	2.75%	*Fusobacterium nucleatum*
0.017%	1.45%	*Prevotella ruminicola*
0.011%	0.99%	*Campylobacter concisus*
0.011%	0.99%	*Bacteroides helcogenes*
0.011%	0.99%	*Mycoplasma hyorhinis*
0.010%	0.84%	*Chlamydia trachomatis*
0.008%	0.69%	*Selenomonas ruminantium*
0.007%	0.61%	*Ralstonia pickettii*
0.007%	0.61%	*Megasphaera elsdenii*
0.006%	0.54%	*Clostridium pasteurianum*
0.006%	0.54%	*Eubacterium rectale*
0.006%	0.54%	*Selenomonas sputigena*
0.006%	0.54%	*Prevotella melaninogenica*
0.006%	0.54%	*Prevotella sp. oral taxon 299*
0.004%	0.38%	*Prevotella denticola*
0.003%	0.31%	*Campylobacter jejuni*
0.003%	0.31%	*Escherichia coli*
0.003%	0.31%	*Streptococcus equi*
0.003%	0.31%	*Roseburia hominis*
0.003%	0.31%	*Coprococcus catus*
0.003%	0.31%	*Coprococcus sp. ART55/1*
0.003%	0.31%	*Lachnoclostridium phytofermentans*
0.003%	0.31%	*[Clostridium] saccharolyticum*
0.003%	0.31%	*Filifactor alocis*
0.003%	0.31%	*Pelotomaculum thermopropionicum*
0.003%	0.31%	*Veillonella parvula*
0.003%	0.31%	*Bacteroides thetaiotaomicron*
0.003%	0.31%	*Bacteroides salanitronis*
0.003%	0.31%	*Prevotella intermedia*
0.003%	0.31%	*Chlamydia muridarum*
0.003%	0.23%	*Clostridium saccharobutylicum*

0.003%	0.23%	*[Eubacterium] eligens*
0.003%	0.23%	*Faecalitalea cylindroides*
0.003%	0.23%	*Mycoplasma hyopneumoniae*
0.002%	0.15%	*Desulfovibrio vulgaris*
0.002%	0.15%	*Proteus mirabilis*
0.002%	0.15%	*[Mannheimia] succiniciproducens*
0.002%	0.15%	*Bradyrhizobium sp. BTAi1*
0.002%	0.15%	*Agrobacterium sp. H13-3*
0.002%	0.15%	*Streptococcus macedonicus*
0.002%	0.15%	*Streptococcus gallolyticus*
0.002%	0.15%	*Lactobacillus reuteri*
0.002%	0.15%	*Lactobacillus amylovorus*
0.002%	0.15%	*Bacillus coagulans*
0.002%	0.15%	*Clostridium cellulovorans*
0.002%	0.15%	*Clostridium beijerinckii*
0.002%	0.15%	*Clostridium kluyveri*
0.002%	0.15%	*Candidatus Arthromitus sp. SFB-rat-Yit*
0.002%	0.15%	*[Ruminococcus] torques*
0.002%	0.15%	*Peptoclostridium difficile*
0.002%	0.15%	*Ruminococcus champanellensis*
0.002%	0.15%	*Faecalibacterium prausnitzii*
0.002%	0.15%	*Acidaminococcus fermentans*
0.002%	0.15%	*Flavobacterium psychrophilum*
0.002%	0.15%	*Pedobacter saltans*
0.002%	0.15%	*Leptotrichia buccalis*
0.002%	0.15%	*Sphaerochaeta pleomorpha*
0.001%	0.08%	*Oscillibacter valericigenes*
0.001%	0.08%	*Ilyobacter polytropus*
0.001%	0.08%	*Sebaldella termitidis*

Supplementary Table 2. Viruses found in metagenomic characterization of *Lawsonia intracellularis* gut homogenate challenge material.

% of Total Reads	% of Viral Reads	Virus
0.005%	20.00%	Elephantid herpesvirus 1
0.005%	20.00%	Dickeya phage RC-2014
0.003%	13.33%	Porcine type-C oncovirus
0.002%	6.67%	Human herpesvirus 7
0.002%	6.67%	Cercopithecine herpesvirus 2
0.002%	6.67%	Glypta fumiferanae ichnovirus
0.002%	6.67%	Trichoplusia ni ascovirus 2c
0.002%	6.67%	Pandoravirus salinus

Supplementary Table 3. PERMANOVA and PERMDISP beta diversity analysis results utilizing the Bray-Curtis dissimilarity and weighted UniFrac distance metrics among Control and Law groups at different time points and intestinal segments.

			PERMANOVA		PERMDISP	
Beta Diversity Metric	DPI	Sample Type	*R*	*P*-value	*F* value	*P*-value
Bray-Curtis	-23	Feces	0.02513	0.361	0.4666	0.4986
Bray-Curtis	0	Feces	0.0777	0.001	0.0265	0.8716
Bray-Curtis	7	Feces	0.0948	0.003	0.4916	0.4881
Bray-Curtis	21	Feces	0.31168	0.001	2.2654	0.1472
Bray-Curtis	28	Feces	0.3004	0.005	0.1303	0.7265
Weighted UniFrac	-23	Feces	0.02781	0.278	0.6409	0.4283
Weighted UniFrac	0	Feces	0.05995	0.06	0.8497	0.3623
Weighted UniFrac	7	Feces	0.0596	0.067	1.337	0.2559
Weighted UniFrac	21	Feces	0.26921	0.001	0.8096	0.3785
Weighted UniFrac	28	Feces	0.27536	0.012	0.9568	0.3536
Bray-Curtis	7	Cecal Digesta	0.11728	0.125	0.129	0.7332
Bray-Curtis	21	Cecal Digesta	0.30653	0.005	0.0183	0.8951
Bray-Curtis	28	Cecal Digesta	0.40582	0.004	1.4128	0.265
Weighted UniFrac	7	Cecal Digesta	0.07972	0.575	0.0841	0.7777
Weighted UniFrac	21	Cecal Digesta	0.34539	0.004	1.6602	0.2266
Weighted UniFrac	28	Cecal Digesta	0.42004	0.004	3.5625	0.09171
Bray-Curtis	7	Ileal Mucosa	0.0822	0.432	0.6664	0.4333
Bray-Curtis	21	Ileal Mucosa	0.21096	0.001	0.9437	0.3567
Bray-Curtis	28	Ileal Mucosa	0.27663	0.007	0.0594	0.8129
Weighted UniFrac	7	Ileal Mucosa	0.08149	0.504	5.3425	0.0434
Weighted UniFrac	21	Ileal Mucosa	0.27011	0.005	0.0002	0.9898
Weighted UniFrac	28	Ileal Mucosa	0.26288	0.013	0.0037	0.9531
Bray-Curtis	7	Ileal Digesta	0.13393	0.046	0.2306	0.6414
Bray-Curtis	21	Ileal Digesta	0.31017	0.011	0.0596	0.8121
Bray-Curtis	28	Ileal Digesta	0.38013	0.004	0.8907	0.3699
Weighted UniFrac	7	Ileal Digesta	0.07904	0.545	0.984	0.7602
Weighted UniFrac	21	Ileal Digesta	0.31696	0.015	0.4898	0.5
Weighted UniFrac	28	Ileal Digesta	0.391	0.002	4.5336	0.0621

Supplementary Table 4. PERMANOVA and PERMDISP beta diversity analysis results utilizing the Bray-Curtis dissimilarity and weighted UniFrac distance metrics among Law and LawVac groups at different time points and intestinal segments.

			PERMANOVA		PERMDISP	
Beta Diversity Metric	DPI	Sample Type	*R*	*P*-value	F value	*P*-value
Bray-Curtis	-23	Feces	0.02514	0.406	0.0019	0.9659
Bray-Curtis	0	Feces	0.04139	0.042	0.1851	0.6694
Bray-Curtis	7	Feces	0.0539	0.023	3.4193	0.0737
Bray-Curtis	21	Feces	0.09629	0.003	0.3548	0.5575
Bray-Curtis	28	Feces	0.14024	0.061	0.0004	0.9906
Weighted UniFrac	-23	Feces	0.0175	0.763	0.009	0.9248
Weighted UniFrac	0	Feces	0.0369	0.168	0.1017	0.7515
Weighted UniFrac	7	Feces	0.03162	0.385	0.7427	0.3952
Weighted UniFrac	21	Feces	0.08131	0.05	1.541	0.2275
Weighted UniFrac	28	Feces	0.15648	0.092	2.634	0.1356
Bray-Curtis	7	Cecal Digesta	0.13067	0.249	0.0647	0.8057
Bray-Curtis	21	Cecal Digesta	0.09051	0.443	1.1289	0.313
Bray-Curtis	28	Cecal Digesta	0.16354	0.024	0.1939	0.6691
Weighted UniFrac	7	Cecal Digesta	0.10363	0.47	0.8683	0.3787
Weighted UniFrac	21	Cecal Digesta	0.05272	0.812	1.0304	0.334
Weighted UniFrac	28	Cecal Digesta	0.15759	0.104	0.0909	0.7693
Bray-Curtis	7	Ileal Mucosa	0.17201	0.024	0.1046	0.7547
Bray-Curtis	21	Ileal Mucosa	0.10175	0.28	0.0861	0.7752
Bray-Curtis	28	Ileal Mucosa	0.20486	0.007	0.2783	0.6094
Weighted UniFrac	7	Ileal Mucosa	0.23874	0.029	0.0187	0.8647
Weighted UniFrac	21	Ileal Mucosa	0.12567	0.134	0.9322	0.3571
Weighted UniFrac	28	Ileal Mucosa	0.21946	0.024	0.0796	0.7836
Bray-Curtis	7	Ileal Digesta	0.12023	0.149	0.1742	0.6862
Bray-Curtis	21	Ileal Digesta	0.08829	0.477	0.5411	0.4789
Bray-Curtis	28	Ileal Digesta	0.14434	0.067	0.0855	0.7759
Weighted UniFrac	7	Ileal Digesta	0.1042	0.395	2.2531	0.1676
Weighted UniFrac	21	Ileal Digesta	0.04561	0.864	0.5277	0.4842
Weighted UniFrac	28	Ileal Digesta	0.15523	0.093	0.0273	0.872

Supplementary Table 5. Differentially abundant genera among LawVac and Law groups at 0 days post infection (21 days post vaccination) found in feces. A negative log2-fold change indicates greater abundance in the Law group. Only significant differences (adjusted *P*-value ≤ 0.05) are shown.

Genus	Log2-Fold Change	Adjusted *P*-value
Coprococcus 1	5.96	3.8E-05
Coprococcus 3	5.88	1.1E-06
Pseudoramibacter	4.63	2.0E-04
Fusobacterium	3.36	2.9E-04
Corynebacterium 1	3.08	3.8E-05
Paraeggerthella	2.91	7.4E-04
Prevotellaceae UCG-004	2.75	1.5E-03
Desulfovibrio	2.74	3.3E-04
Eggerthella	2.64	1.1E-05
Unknown Chloroplast	2.56	1.8E-04
Holdemania	2.55	1.2E-03
Butyrivibrio	2.36	4.2E-03
Lysobacter	2.16	3.8E-05
Acidaminococcus	2.10	6.4E-05
Catenibacterium	2.07	3.2E-02
Olsenella	2.06	7.2E-03
Ruminococcaceae UCG-011	2.01	3.0E-03
Sphingobium	1.99	1.2E-04
Staphylococcus	1.98	3.4E-05
Unknown BIrii41	1.95	6.4E-05
Allisonella	1.94	2.5E-04
Odoribacter	1.94	1.3E-04
Dyella	1.91	7.1E-05
Alistipes	1.87	1.6E-02
Ruminococcaceae UCG-008	1.86	3.1E-02
Unknown Bacteroidetes	1.83	9.5E-03
Anaerovorax	1.80	3.8E-05
Unknown Clostridia	1.80	1.2E-03
Anaerococcus	1.75	2.6E-04
Anaerovibrio	1.72	2.6E-03
Unknown Coriobacteriales	1.67	1.3E-02
Devosia	1.66	6.0E-04
Shuttleworthia	1.65	3.7E-02
Elusimicrobium	1.64	6.7E-04
Marvinbryantia	1.63	7.0E-03
Negativibacillus	1.61	5.3E-03
GCA-900066225	1.59	7.2E-03

Pseudomonas	1.43	9.2E-04
Luteolibacter	1.42	3.0E-04
Cloacibacillus	1.41	6.7E-04
Actinomyces	1.40	9.0E-05
Unknown Lactobacillales	1.39	4.1E-03
Anaerofilum	1.39	5.8E-04
Ruminococcaceae UCG-004	1.39	1.6E-02
Unknown Burkholderiaceae	1.36	7.2E-03
Parasutterella	1.26	1.5E-02
Lawsonia	1.26	1.6E-02
Streptococcus	1.16	2.5E-02
Megasphaera	1.13	4.9E-02
Enterorhabdus	0.96	4.6E-02
Ruminiclostridium 5	0.94	4.9E-02
Sutterella	0.90	1.5E-02
Erysipelotrichaceae UCG-002	-1.15	7.0E-03
Treponema 2	-1.57	3.8E-02
Ruminobacter	-1.69	9.0E-05
[Eubacterium] ventriosum group	-1.80	1.3E-03
Intestinibacter	-1.84	1.3E-03
Unknown p-251-o5	-1.93	1.1E-05
Mitsuokella	-2.04	2.5E-03
[Eubacterium] oxidoreducens group	-2.04	2.7E-02
Clostridium sensu stricto 1	-2.11	2.1E-02
Acinetobacter	-2.17	2.2E-04
Ruminococcaceae UCG-009	-2.31	1.1E-03
Candidatus Saccharimonas	-2.32	7.2E-06
Rikenellaceae RC9 gut group	-2.43	6.2E-03
Ruminococcaceae UCG-003	-2.54	7.1E-05
Unknown Rikenellaceae	-2.72	5.8E-04
Family XIII UCG-001	-2.83	1.0E-04
Turicibacter	-3.17	2.2E-04
Unknown T34	-3.22	4.9E-05
Akkermansia	-3.77	1.0E-04
Muribaculum	-5.47	1.1E-05
dgA-11 gut group	-6.54	5.4E-07

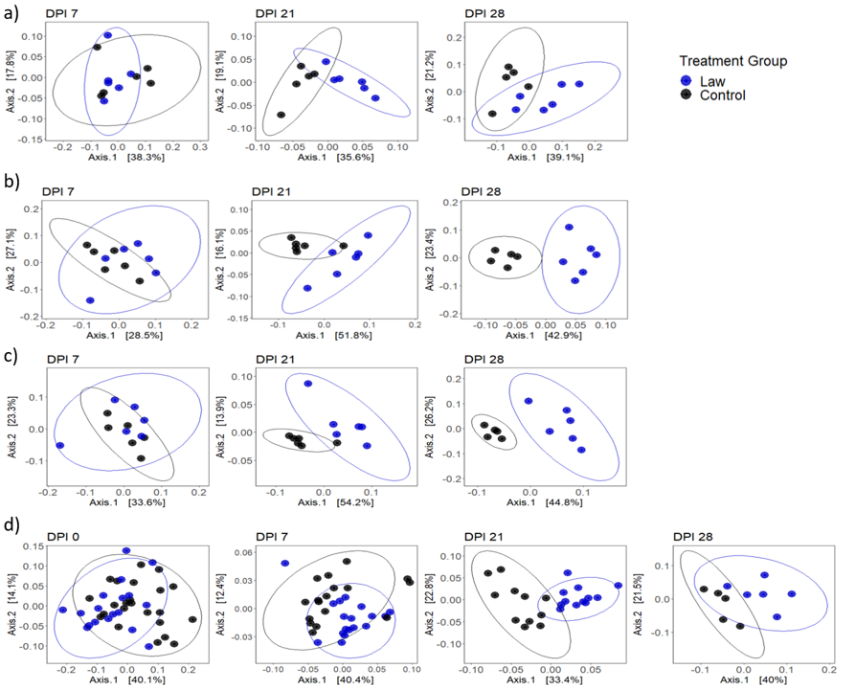

Supplementary Figure 1. Principal coordinate analysis (PCoA) plots of Weighted UniFrac distance among Law and Control groups in a) ileum mucosal samples; b) ileal digesta samples; c) cecal digesta samples; d) fecal samples. DPI, days post infection.

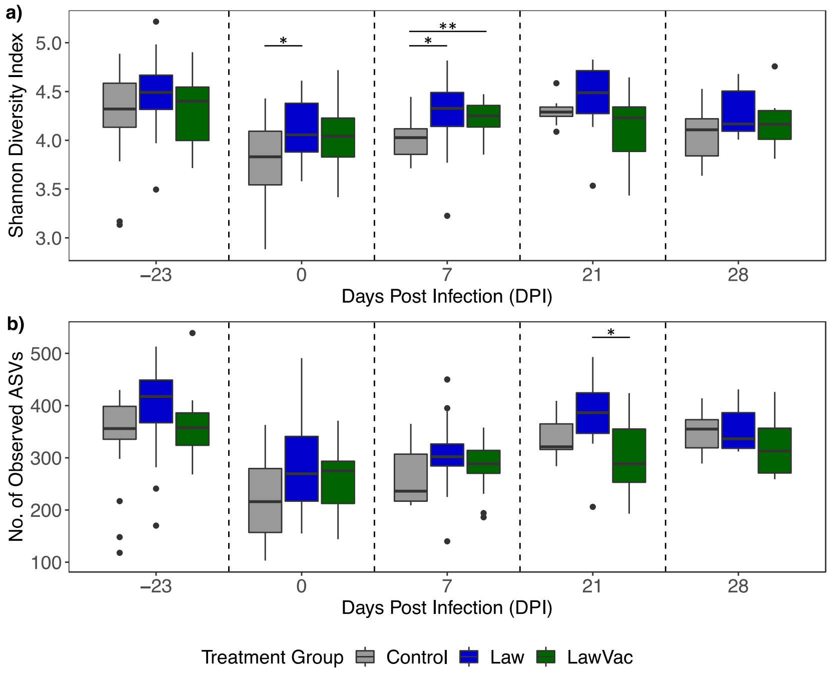

Supplementary Figure 2. Alpha diversity analysis among different treatment groups in fecal samples evaluated by a) Shannon diversity index and b) number of observed ASVs. Adjusted *P*-values: *, $P \leq 0.05$; $P \leq 0.01$.

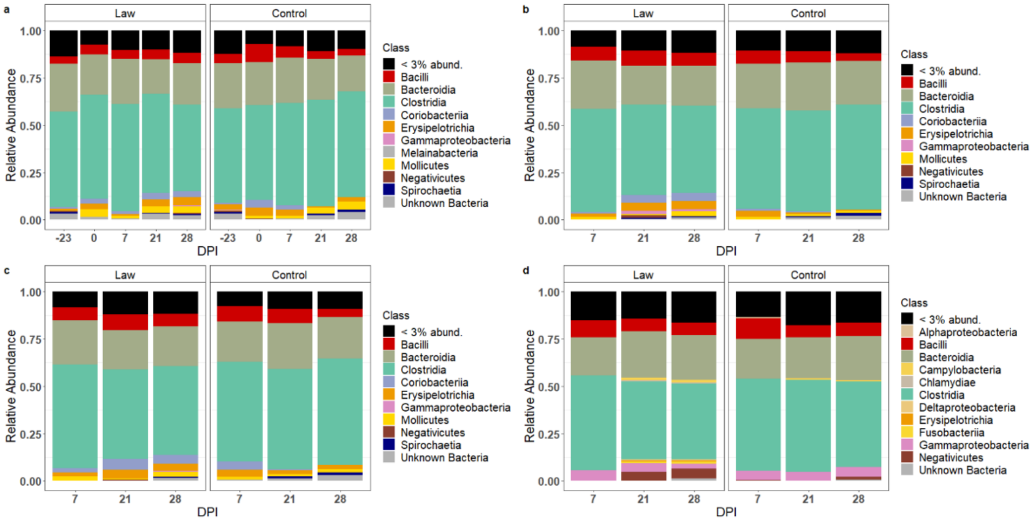

Supplementary Figure 3. Relative abundance of different bacterial classes among a) feces; b) cecal digesta; c) ileal digesta; d) ileal mucosa. DPI, days post infection.

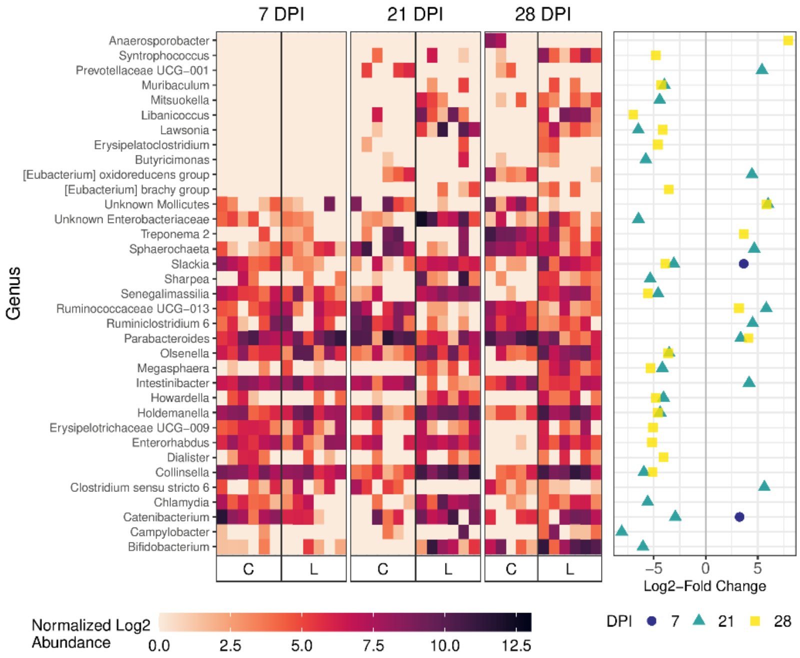

Supplementary Figure 4. Differential abundance of microbial genera in ileal digesta samples between the Law and Control groups at 7, 21, and 28 dpi. The 20 genera with the largest significant log2-fold change at each dpi are included. The heatmap shows the CSS-normalized log2 abundance of each genera per sample, with the significant log2-fold changes by dpi plotted on the right panel. Note that a genus only has to be one of the top 20 significantly differentially abundant at one dpi for it to be included in the figure. A positive log2-fold change indicates greater abundance in the Control group and a negative log2-fold change indicates greater abundance in the Law group.

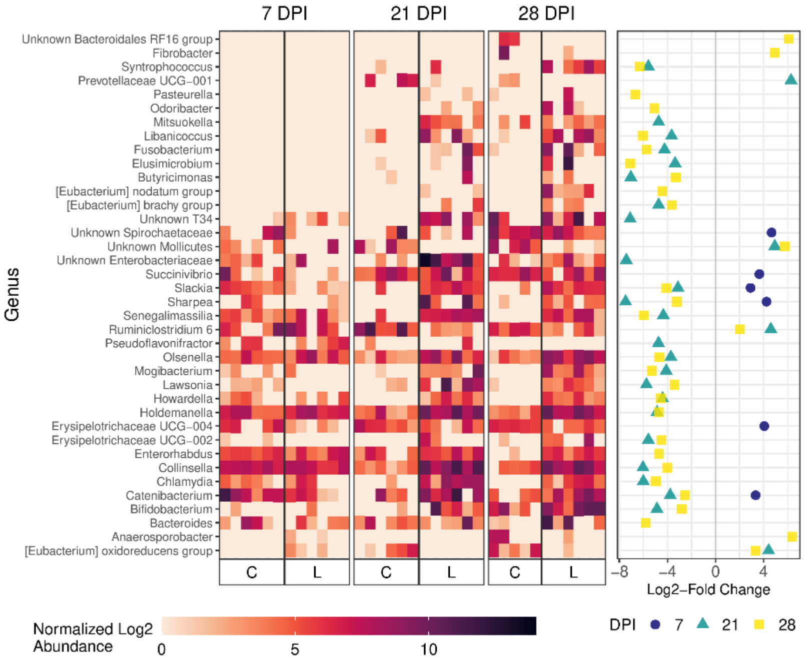

Supplementary Figure 5. Differential abundance of microbial genera in cecal digesta samples between the Law and Control groups at 7, 21, and 28 dpi. The 20 genera with the largest significant log2-fold change at each dpi are included. The heatmap shows the CSS-normalized log2 abundance of each genera per sample, with the significant log2-fold changes by dpi plotted on the right panel. Note that a genus only has to be one of the top 20 significantly differentially abundant at one dpi for it to be included in the figure. A positive log2-fold change indicates greater abundance in the Control group and a negative log2-fold change indicates greater abundance in the Law group.

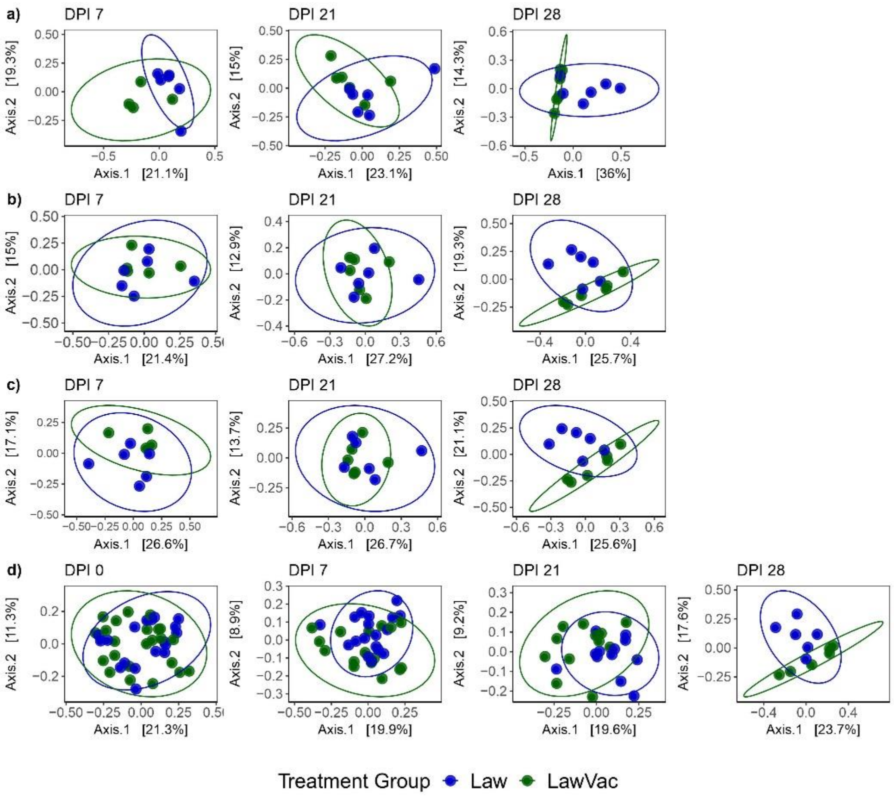

Supplementary Figure 6. PCoA plots using Bray-Curtis dissimilarity of samples from a) ileal mucosa; b) ileal digesta; c) cecal digesta; d) feces among Law and LawVac groups at different days post infection (DPI). Ellipses represent the 95% confidence level of the multivariate Student's t-distribution.

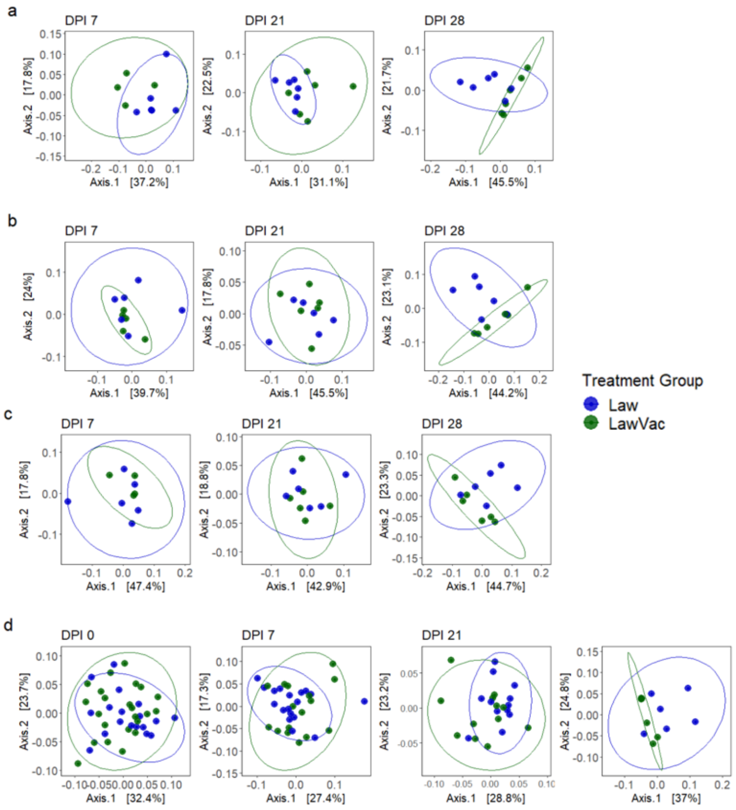

Supplementary Figure 7. PCoA plots using weighted UniFrac distance of samples from a) ileal mucosa; b) ileal digesta; c) cecal digesta; d) feces among Law and LawVac groups at different days post infection (DPI). Ellipses represent the 95% confidence level of the multivariate Student's t-distribution.

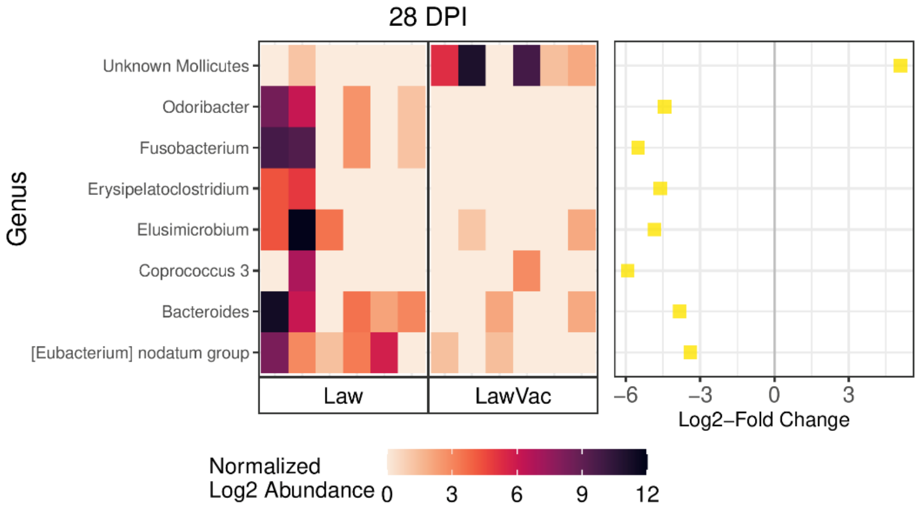

Supplementary Figure 8. Differential abundance of microbial genera in ileal digesta samples between the Law and LawVac groups at 28 dpi. The heatmap shows the CSS-normalized log2 abundance of each genera per sample, with the significant log2-fold changes plotted on the right panel. A positive log2-fold change indicates greater abundance in the LawVac group and a negative log2-fold change indicates greater abundance in the Law group.

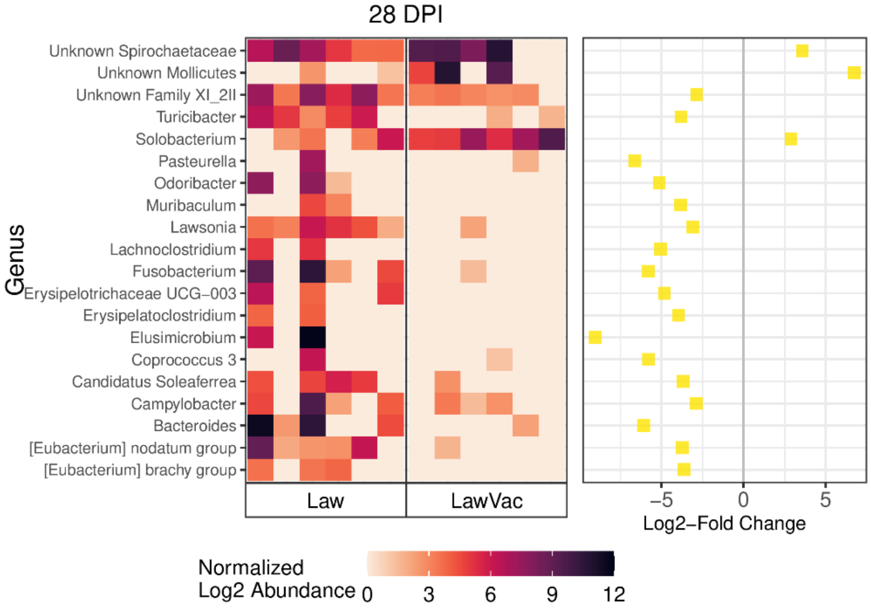

Supplementary Figure 9. Differential abundance of microbial genera in cecal digesta samples between the Law and LawVac groups at 28 dpi. The 20 genera with the largest significant log2 fold change are included. The heatmap shows the CSS-normalized log2 abundance of each genera per sample, with the significant log2-fold changes plotted on the right panel. A positive log2-fold change indicates greater abundance in the LawVac group and a negative log2-fold change indicates greater abundance in the Law group.
